# The Medical Congress

**Published:** 1885-10

**Authors:** 


### THE MEDICAL CONGRESS.

A meeting of the general committee of the International Medical Congress was held in New York City, on the 3d ult., and some of the changes necessary to success were made. Whether these were sufficiently radical to secure the co-operation of the men without whose aid there cannot be anything like a full representation of the medical profession of the United States, is exceedingly problematical. Unless there can be entire harmony and unity of purpose, the proposed meeting will prove anything but a blessing. The doors of admission have been more widely opened, and there will be no attempt to exclude those who are not representatives of, or represented in, the American Medical Association. But all such are rigidly debarred from holding any position of honor or trust in the meeting.

The principal bone of contention, however, still remains, and that is, whether or not the Congress shall be exclusively managed and controlled by a single society; whether or not, it shall be in effect but an enlarged meeting of that society. This we take to be the key-note of all the contention that has been aroused. All the foreign members are agreed that this was not anticipated in the acceptance of the American invitation, that it is entirely foreign to the aims and past history of the Congress, and that it is establishing a dangerous precedent. It is greatly to be regretted that the committee, at its late meeting, did not unmistakably recede from this ground. Had this been done, no mere question of personality would have been allowed to interfere with the success of the meeting. Any exhibition of private spleen, or of personal ambition, would have been sternly frowned down by the whole profession, and all would, by the force of public opinion—the question of principle having been eliminated—be compelled either to a cordial co-operation, or to a quiet acceptance of the situation. But we fear that the essential step was not taken ; that the powerful opposition is not disarmed, and that their influence will be so felt that the meeting will either be called for Berlin, or will be almost entirely neglected by the foreign members, without whose presence it will be a scientific failure.

The committee has receded from its decision abolishing the section of Oral and Dental Surgery, and that has been restored, and Dr. Taft again named as its chairman. If, now, the jarring elements can be harmonized, and the medical profession so united that success is possible, there is no reason why dentists should not go to work with a will to make their section as great a triumph as was that of 1881. But if the fight is to be kept up, and the meeting be only a battle-ground for warring factions, the less we have to do with it the better. In our opinion, the proper attitude of dentists, for the present, is one of expectancy—of waiting to see what will be the outcome of the whole matter.

